# Hospitalization and post-discharge care in South Africa: A critical event in the continuum of care

**DOI:** 10.1371/journal.pone.0208429

**Published:** 2018-12-13

**Authors:** Cody Cichowitz, Rachael Pellegrino, Katlego Motlhaoleng, Neil A. Martinson, Ebrahim Variava, Christopher J. Hoffmann

**Affiliations:** 1 Johns Hopkins School of Medicine, Baltimore, MD, United States of America; 2 Perinatal HIV Research Unit, Gauteng, South Africa; 3 Department of Medicine, Tshepong Hospital, Klerksdorp, South Africa; 4 Division of Infectious Disease, Johns Hopkins University School of Medicine, Baltimore, MD, United States of America; Yokohama City University, JAPAN

## Abstract

**Objectives:**

The purpose of this prospective cohort study is to characterize the event of acute hospitalization for people living with and without HIV and describe its impact on the care continuum. This study describes care-seeking behavior prior to an index hospitalization, inpatient HIV testing and diagnosis, discharge instructions, and follow-up care for patients for patients being discharged from a single hospital in South Africa.

**Methods:**

A convenience sample of adult patients was recruited from the medical wards of a tertiary care facility. Baseline information at the time of hospital admission, subsequent diagnoses, and discharge instructions were recorded. Participants were prospectively followed with phone calls for six months after hospital discharge. Descriptive analyses were performed.

**Results:**

A total of 293 participants were enrolled in the study. Just under half (46%) of the participants were known to be living with HIV at the time of hospital admission. Most participants (97%) were given a referral for follow-up care; often that appointment was scheduled within two weeks of discharge (64%). Only 36% of participants returned to care within the first month, 50% returned after at least one month had elapsed, and 14% of participants did not return for any follow up.

**Conclusions:**

Large discrepancies were found between the type of post-discharge follow-up care recommended by providers and what patients were able to achieve. The period of time following hospital discharge represents a key transition in care. Additional research is needed to characterize patients’ risk following hospitalization and to develop patient-centered interventions.

## Introduction

Prior to the availability of antiretroviral therapy (ART) for HIV, hospitals in many countries in sub-Saharan Africa (SSA), were crowded with people with advanced HIV and associated opportunistic infections including cryptococcal meningitis, toxoplasmosis, chronic diarrhea, and disseminated tuberculosis (TB) [1]. Due to the rapid expansion of HIV programs and increased access to ART, individuals living with HIV can have long lifespans and may be at risk for traditional illnesses of aging [2]. Concurrently, HIV-associated mortality has declined from peak levels in 2006 and the burden of disease in medical wards has shifted [3]. Given that a majority of patients survive medical hospitalization and are discharged with complex medical needs [4–7], there is an emerging need to understand the impact of acute hospitalization and the subsequent transition back to community-based care for people living with HIV and those without.

In high-resource settings the event of acute hospitalization and care transitions have been well studied for a variety of disease states [8–14]. These events have been associated with poor outcomes [15,16], and a diverse set of interventions has been developed to increase support during this time, including multi-disciplinary care coordination and discharge planning, home visits, follow-up phone calls, and health systems interventions like increased access to specialty clinics or a packaging of services offered at one clinic visit [8–14]. In resource-limited settings, these types of interventions have not yet been implemented on a large scale and data are needed to characterize the needs of hospitalized patients and to develop effective evidenced-based interventions for follow-up care.

In countries with a high burden of HIV, like South Africa, medical admissions provide important information about the care-seeking behaviors of people prior to hospitalization and highlight gaps in both community-based and hospital-based HIV testing and ART initiation. Increasing the rate of inpatient HIV testing has already been identified as an important need [17,18], and it is thought that HIV-associated mortality persists in large part due to delayed presentation to care for people living with HIV, low rates of community- and hospital-based HIV testing, failed linkage to care, and low retention in ART programs among people living with HIV [7,19,20]. Improving the continuity of care and retention in chronic disease (and HIV) programs is a key step in realizing the potential individual and public health benefits of improved access to care and ART [7,21].

Currently, little is known about the impact of the event acute hospitalization on the continuum of care in SSA. The purpose of this prospective cohort study was to explore characteristics of patients discharged from an adult medical ward in South Africa, and compare care-seeking behaviors prior to the index hospitalization, discharge instructions, and subsequent follow-up care for patients with and without HIV.

## Materials and methods

### Study setting

This prospective cohort study took place in the North West province of South Africa. A convenience sample was recruited from the adult medical wards of a government referral and tertiary care hospital. The hospital serves the local metropolitan area and surrounding peri-urban and rural areas. The adult medicine department consists of 216 beds and averages 30 admissions per day. Participants were recruited from August 2014 to December 2014 and follow-up data collection continued until August 2015. At that time, South African HIV guidelines stipulated an ART initiation threshold at a CD4 cell count of 350 cells/mm^3^, which increased to 500 cells/mm^3^ during the study period.

### Participants

Adult (≥ 18 years) patients admitted to the medical ward were eligible for study enrollment, provided that they or their legally authorized representative were able to provide informed consent, and spoke one of the four study languages (English, Setswana, isiXhosa, or isiZulu).

### Study procedures

Monday through Thursday mornings, trained research staff went to the medical wards and made a general announcement about the study, introducing patients to the goals and design of the research project. Study staff would then individually approach each patient and ask if they were willing to participate. Participants who enrolled were given 20 ZAR (~2 dollars) of mobile phone airtime at enrollment, in keeping with local remuneration rates, and provided an additional 20 ZAR of airtime at each additional time point. Between 3 to 5 participants were enrolled daily.

After completing the informed consent process, study staff recorded the participants’ contact information and completed a detailed enrollment survey, which included basic demographic information, past medical history, care utilization prior to hospitalization, and access to medical care. For those participants with known HIV at the time of hospital admission, specific information was captured regarding their HIV care, including ART status, self-reported adherence, and care utilization prior to the index admission.

At the time of hospital discharge, research assistants captured all discharge diagnosis documented in the medical record, the results of any laboratory testing conducted during the admission, and the physicians’ recommendations for follow-up care. If a participant died prior to discharge, they were removed from the study.

### Participant follow-up

At the time of study enrollment, the study team recorded the participant’s primary phone number and the contact information of up to three family members, friends, or alternative contacts. Place of employment and home address were also recorded. The study team attempted to contact the participant 4–8 weeks, 3 months, and 6 months after discharge. At each contact point following discharge, the study team captured vital status (alive or dead), and care utilization patterns including any appointments attended and hospital admissions.

For each scheduled contact, the study staff attempted up to five different phone calls (of all recorded numbers) on different days of the week (including weekends) and different times of the day. If a participant returned to the hospital either for an appointment or was readmitted, the study team was informed and collected any follow-up data indicated at that time. At the end of data collection, a hospital medical record audit was performed for any participant who had not been reached during the follow-up period. The audit assessed for attendance at hospital-based clinic appointments, readmission at the tertiary care hospital, and recorded death.

### Data analysis

The analysis presented is descriptive in nature and data are presented as proportions, percentages, and medians with interquartile ranges. Bivariate testing was conducted to compare differences in participant characteristics, care utilization prior to hospitalization, and access to care between participants known to be living with HIV at the time of admission and those who were HIV negative or had not been tested. Similarly, bivariate testing was used to compare differences in discharge instructions, follow up-care, and six-month outcomes between those known to be living with HIV at discharge and those who were HIV negative or had not been tested. Study data are provided in S1 File.

### Ethics

This research was conducted according to the principles expressed in the Declaration of Helsinki; written or witnessed oral informed consent was obtained from all participants prior to study procedures. The study was approved by the institutional review boards of the Johns Hopkins University School of Medicine and the University of the Witwatersrand.

## Results

This prospective cohort study recruited 298 people upon admission to the adult medical ward and follow-up was continued for six months after discharge. Five participants died prior to hospital discharge and were removed from the study. Participant characteristics, care utilization prior to hospitalization, and access to care are described in Table 1. Among those surviving to discharge, 47% (137/293) were female and the median age was 37.5 years (interquartile range [IQR] 29, 48). A little less than half (46% [n = 136]) of the 293 participants recruited had been living with HIV prior to hospital admission. Care utilization was high prior to hospitalization; approximately half (51%; 148/293) of patients reported visiting a clinic during the 30 days prior to hospital admission. People known to be living with HIV at the time of admission accessed care more frequently than those who were HIV negative or of unknown status (60% vs. 43% accessed care within 30 days of hospital admission; p = 0.04). A little less than a quarter (23%; 68/293) of the participants had been admitted to the hospital within the six months preceding the index admission, and 9% (27/293) had been admitted within 30 days prior to the index admission. A little less than half (45%; 131/293) of the participants reported difficulty accessing outpatient care (51% in PLHIV vs. 42% in HIV negative individuals; p = 0.03). A majority of people could walk to the local clinic (68%; 198/293). Few people (17%; 51/293) could walk to the hospital or specialty clinics; most had to pay for transportation.

**Table 1:** Participant characteristics, care utilization prior to hospitalization, and access to care.

	All Patients (n = 293)		HIV positive at admission (n = 136)		HIV negative or unknown at admission (n = 157)		P-value*
	n	%	n	%	n	%	
**Characteristics**							
Age—median (Q1, Q3)	37.5 (29, 48)		37 (31, 45)		38 (28, 51)		.76
Sex							
Female	137	47%	75	55%	62	39%	.007
Male	156	53%	61	45%	95	61%	
**Care utilization**							
Clinic attendance							
Within 6 months							
No	100	34%	37	27%	63	40%	.02
Yes	193	66%	99	73%	94	60%	
Within 30 days							
No	145	49%	55	40%	90	57%	.004
Yes	148	51%	81	60%	67	43%	
Hospital admission							
Within 6 months							
No	225	77%	104	76%	121	77%	.90
Yes	68	23%	32	24%	36	23%	
Within 30 days							
No	266	91%	124	91%	142	90%	.83
Yes	27	9%	12	9%	15	10%	
**Access to care**							
Difficulty accessing care							
No	162	55%	66	49%	96	61%	.03
Yes	131	45%	70	51%	61	39%	
Access to primary care							
Walks	198	68%	95	70%	103	66%	.44
Pays for transport	95	32%	41	30%	54	34%	
Access to tertiary care							
Walks	51	17%	18	13%	33	21%	.08
Pays for transport	242	83%	118	87%	124	79%	

*P-values were generated using Chi-squared testing for dichotomous variables and Mann–Whitney U (rank-sum) testing for continuous variables.

For the 136 people who were known to be living with HIV at the time of admission, a majority had been living with HIV for over one year (65%) (Table 2). Over half the PLHIV reported being on ART (88/136), and three quarters (75% [n = 66]) of 88 participants on ART had been taking medicine for over a year. 24/88 (27%) participants on ART self-reported missing medication within the month prior to hospitalization.

**Table 2:** HIV care prior to hospitalization (n = 136).

	n	%
**Time living with HIV**		
0–6 months	19	14%
6 months—1 year	12	9%
1 year—5 years	50	37%
> 5 years	39	29%
Missing	16	12%
**Last HIV appointment**		
Does not attend	41	30%
0–1 month	69	51%
1–2 months	17	13%
> 2 months	9	7%
**ART**		
Yes	88	65%
**Time on ART (n = 88)**		
0–6 months	12	14%
6 months—1 year	10	11%
1–5 years	34	39%
> 5 years	31	35%
Missing	1	1%
**Self-reported ART adherence (n = 88)**		
Missed pills in last 30 days	24	27%

Of the 157 patients eligible for HIV testing during admission (i.e. not known to be living with HIV), 32 (20%) tested positive for HIV, 68 (43%) tested negative for HIV, and 57 (36%) were not tested. Of the 32 participants diagnosed with HIV during the index admission, the median CD4 count was 56 cells/mm^3^ (IQR: 23, 372) and none were started on ART prior to hospital discharge. Of the 136 PLHIV with known HIV prior to admission, the median CD4 count was 182 cells/mm^3^ (IQR 35, 383). Of the 136 participants known to be living with HIV at the time of admission, 88 (65%) were on ART at the time of admission and continued after discharge, 38 (28%) were not on ART at the time of admission and were not initiated on ART, and 10 (7%) were started on ART for the first time.

**Fig 1:**
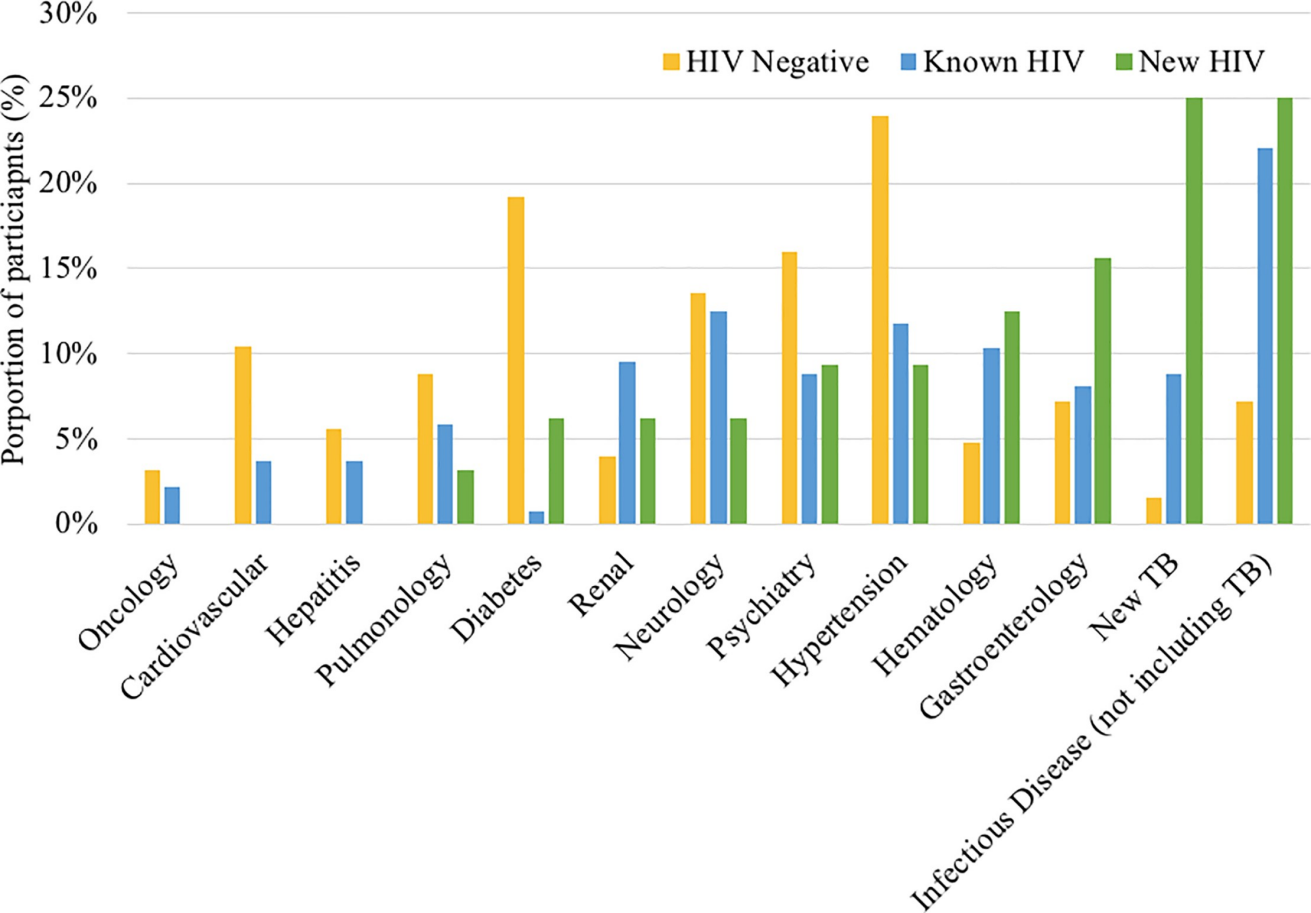
Discharge diagnoses by HIV status.

For the 32 participants diagnosed with HIV during hospitalization, a new case of TB disease (25%), infectious disease excluding TB (25%), or gastrointestinal disorder (16%) were the most common discharge diagnoses (Fig 1). For the people with known HIV at the time of admission, new infections excluding TB (22%), neurologic disorders (13%) and hypertension (12%) were the most common discharge diagnoses. Finally, among those without HIV, hypertension (24%), diabetes (19%), and psychiatric disorders (16%) were the main causes of admission.

Most participants (97%; 263/270) were given a referral for follow-up care (Table 3), with the most common referral to return to the tertiary care facility for specialty ambulatory care (83%; 207/250). Over half (64%; 150/234) were requested to return to the facility within two weeks of discharge.

**Table 3:** Discharge instructions, care engagement, and six-month outcomes.

	All Patients (n = 293)		HIV positive at discharge (n = 168)		HIV negative or unknown at discharge (n = 125)		P-value*
	n	%	n	%	n	%	
Follow up appointment scheduled							
No	7	3%	2	1%	5	4%	.14
Yes	263	97%	153	99%	110	96%	
Missing	23	-	13	-	10	-	
Type of follow up							
Tertiary care	207	83%	115	79%	92	88%	.09
Primary care	43	17%	30	21%	13	12%	
No appointment given	7	-	2	-	5	-	
Missing	36	-	21	-	15	-	
Scheduled time from discharge to first follow up appointment							
0–2 weeks	150	64%	90	67%	60	61%	.51
3–4 weeks	73	31%	40	30%	33	33%	
> 1 month	11	5%	5	4%	6	6%	
No appointment given	7	-	2	-	5	-	
Missing	52	-	31	-	21	-	
Actual time from discharge to first follow up appointment							
0–2 weeks	43	18%	23	16%	20	20%	.77
3–4 weeks	44	18%	24	17%	20	20%	
> 1 month	119	50%	73	52%	46	46%	
Never attended	34	14%	20	14%	14	14%	
No appointment given	7	-	2	-	5	-	
Missing	46	-	26	-	20	-	
6 month outcomes							
Unreachable	62	21%	32	19%	30	24%	.78
Unreachable after 3 m	20	7%	12	7%	8	6%	
Alive	163	56%	95	57%	68	54%	
Readmission or death	48	16%	29	17%	19	15%	
Readmission	29	-	15	-	14	-	
Death	19	-	14	-	5	-	

*P-values were generated using the Fisher exact text.

Following discharge, only 36% (87/240) of participants returned for a clinic visit within one month of discharge, 50% (119/240) returned over a month after discharge, and 14% (34/240) of participants did not return for any follow-up appointment. The proportion of patients with scheduled follow-ups and those that returned did not differ by HIV status. Six months after discharge, 56% (163/293) of patients were confirmed to be alive, 21% (62/293) of participants were unreachable during the entire follow-up period and 7% (20/263) were not reachable by three months after hospital discharge. Of those with successful contact or outcome ascertainment, 29/231 (13%) had been readmitted at least once, and 19/231 (8%) were known to have died. Outcomes, including death, readmission, and loss to study follow-up, did not differ by HIV status.

## Discussion

This prospective cohort study recruited 293 patients from the adult medical wards at a tertiary referral hospital, found a high rate of care utilization prior to hospital admission, a large proportion of previously undiagnosed HIV, low rates of inpatient HIV testing and ART initiation, and low rates of ambulatory care follow-up as directed by hospital discharge instructions.

The coverage of inpatient HIV testing and ART initiation in this study has important implications and highlights a key challenge associated with the implementation of large-scale HIV-programs. At the time of this study, South African guidelines stipulated that HIV testing should be offered to all patients in health facilities using an opt-out approach [22]. A recent systematic review and meta-analysis of HIV testing in sub-Saharan Africa found a pooled coverage of provider initiated or opt-out testing in healthcare facilities to be 18% [18]. In the review, provider-initiated, facility-based testing typically identified people with lower CD4 and symptomatic disease, similar to the participants in this study. The findings of this study reaffirm that inpatient wards remain an important venue for the delivery of key elements of HIV programing, including HIV testing, ART initiation, and adherence counseling. Missed opportunities to improve HIV-related outcomes in hospitalized patients may lead to unnecessary readmissions and increased morbidity and mortality. These data suggest a need for implementation science-based interventions to improve facility-based testing and improve provider adherence to national guidelines. This is particularly relevant, given South Africa’s recent adoption of universal test and treat and push toward the UNAIDS 90-90-90 goals. Improving hospital-based testing and ART initiation may help identify a particularly vulnerable population of people living with advanced HIV and support progress toward the aforementioned targets [17].

In this study, acute hospitalization represented an important juncture in the care continuum, both for those living with and without HIV. Prior to the index hospitalization, many participants reported receiving community-based care or being admitted to the hospital within the preceding six months. These interactions with the health care system may represent a missed opportunity to prevent hospitalization through treatment modifications, increased support, and earlier diagnosis of HIV and TB. Recently, much attention has been placed on developing differentiated models of care that can effectively focus resources on the patients that need them the most and simplify management for patients that are stable and healthy [23–25]. Within this sample, there is a subset of high-utilizing patients who likely would benefit from a different package of community-based interventions than patients receiving routine care for stable conditions. A new paradigm is needed for the management of chronic conditions like HIV that mobilizes resources for patients who do not need acute inpatient treatment but would benefit from closer monitoring than can be provided at most community-based primary care clinics. Policy interventions are needed to incentivize cost-effective, networked systems of health care that include primary care, intermediate care, home visits, and hospital and specialized care.

At the time of discharge, almost all patients were referred for follow-up care and most to specialized care. However, there was a clear discordance between the type of care providers considered appropriate and what the participants were actually able to do. Over half of participants did not return for care within the first two months despite it being recommended. There are several possible explanations for this. First, at baseline, a significant number of participants reported difficulty accessing care and a majority reported that they were unable to walk to the tertiary care facility. Second, it is also possible that in the weeks following discharge, patients were still too ill and/or weak to travel back to the hospital for specialized care. Regardless, the combination of high repeat inpatient utilization and failure to successfully follow-up with outpatient care emphasizes the need to develop patient-centered approaches to post-hospital health management [26,27]. While a modest number of deaths and readmissions were captured and the data were largely consistent with previous reports [28–31], we hypothesize that mortality and readmission rates were higher, especially given the large number of participants that were unreachable. The study was limited in that it consisted of a convenience sample, recruited from a single center in the North West province of South Africa. The general population and population of hospital patients across South Africa may differ from this sample. Additionally, the health care system and delivery of care may vary significantly across institutions and geographic locations within the country.

This study captured important discontinuities in care as patients transition home following acute hospitalization. This period of time appears to be associated with disengagement from care and also appeared to be associated with increased morbidity and mortality. Additional research is needed to characterize patients’ risk and vulnerability after hospitalization, retention in HIV and other chronic disease programs following an inpatient stay, integrate services across the care-continuum, and pilot patient-centered interventions to improve health outcomes for those being discharge from acute care hospital stays.

## Supporting information

S1 File Study dataset. (CSV)
